# A retrospective analysis of uropathogens isolated and antimicrobial susceptibility patterns at a regional hospital in North West province, South Africa

**DOI:** 10.4102/ajlm.v14i1.2845

**Published:** 2025-11-30

**Authors:** Miguel J. Teixeira, Vian Pretorius, Robert C.J.G. Hunt, Sanam Morar, Jamie L. Colloty, Caleb M. Radebe, Rajen Morar

**Affiliations:** 1 Ampath Allergy Clinic, Pretoria, South Africa; 2 Department of Internal Medicine, Chris Hani Baragwanath Academic Hospital, Johannesburg, South Africa; 3 Department of Internal Medicine, Klerksdorp-Tshepong Complex Academic Hospital, Klerksdorp, South Africa; 4 Department of Chemical Pathology, Faculty of Health Sciences, University of Pretoria, Pretoria, South Africa; 5 Department of Chemical Pathology, Faculty of Health Sciences, National Health Laboratory Service, Tshwane Academic Division, Pretoria, South Africa; 6 Department of Microbiology, Faculty of Health Sciences, National Health Laboratory Service, Tshwane Academic Division, Pretoria, South Africa; 7 Department of Medical Microbiology, Faculty of Health Sciences, University of Pretoria, Pretoria, South Africa; 8 Department of Internal Medicine, Faculty of Health Sciences, Potchefstroom Hospital, Potchefstroom, South Africa; 9 Department of Internal Medicine, Faculty of Health Sciences, School of Medicine, University of the Witwatersrand, Johannesburg, South Africa; 10 Department of Internal Medicine, Faculty of Health Sciences, Charlotte Maxeke Johannesburg Academic Hospital, Johannesburg, South Africa

**Keywords:** urinary tract infections, antimicrobial resistance, *Escherichia coli*, nitrofurantoin, ciprofloxacin

## Abstract

**Background:**

Urinary tract infections are among the most common infections affecting the general population. Their high incidence, as well as frequent antimicrobial use, contribute significantly to the development of antimicrobial resistance (AMR).

**Objective:**

To determine the profile and prevalence of uropathogens isolated from urine specimens at a regional hospital and assess susceptibility patterns to commonly used antimicrobials recommended by the National Essential Medicines List (NEML).

**Methods:**

This was a retrospective evaluation of laboratory reports for all urine specimens submitted between 01 January 2020 and 31 December 2023.

**Results:**

The most frequently cultured organisms were *Escherichia coli* (*n* = 1481; 42%); *Klebsiella pneumoniae* (*n* = 568; 16%); *Enterococcus faecalis* (*n* = 249; 7%); *Proteus mirabilis* (*n* = 229; 7%), *Enterobacter cloacae* (*n* = 137; 4%), and *Candida albicans* (*n* = 119; 3%). *Escherichia coli* maintained high sensitivity to antimicrobials such as nitrofurantoin (92.2%) and gentamicin (90.6%), whilst *K. pneumoniae* had decreased sensitivities of 40% and 77%, respectively. Cumulative sensitivities of commonly used first-line antimicrobials showed low rates of susceptibility to ciprofloxacin (77%), nitrofurantoin (67%), and amoxicillin/clavulanate (68.7%).

**Conclusion:**

*Escherichia coli* was the most commonly identified isolate and remains sensitive to nitrofurantoin. It was, however, resistant to ciprofloxacin, amoxicillin/clavulanate, and trimethoprim sulfamethoxazole, as were all the other Gram-negative organisms. These sensitivity patterns do not align with the antimicrobials recommended in the current NEML guidelines, and highlight the need for targeted therapy and interventions.

**What this study adds:**

This retrospective analysis identifies predominant uropathogens’ updated antimicrobial susceptibility profiles, some of which misalign with NEML guidelines. Insights will guide targeted antimicrobial stewardship, empiric therapy, and local surveillance to curb AMR.

## Introduction

Urinary tract infections (UTIs) represent a significant health concern globally, affecting an estimated 404.6 million individuals in 2019, and contributing to 5.2 million disability-adjusted life years.^1^ A systematic review of the aetiology and prevalence in sub-Saharan Africa found the prevalence to be 32.1%, with South Africa recording the highest prevalence at 67.1%.^2^ Women have a higher incidence compared with men; in their lifetime, approximately 50% of women will be diagnosed with a UTI, with 20% – 40% experiencing recurrences.^3^ Globally, the most common causative organisms of community-acquired UTIs are Gram-negative bacteria, predominantly *Escherichia coli* and *Klebsiella pneumoniae*.^4^

The high incidence of a positive urinary culture frequently results in the prescription of an antimicrobial, which is often unnecessary, as in the case of asymptomatic bacteriuria. This has the effect of increasing the antimicrobial load in the environment, and exacerbation of the pandemic of antimicrobial resistance (AMR), which has steadily increased internationally because of over- and misuse of antimicrobials, both in humans and animals.^5^ Concurrently, the development of novel antimicrobial therapies has lagged significantly behind the development of AMR, highlighting the need for intensification of antimicrobial stewardship.^5,6^

A recent systematic analysis of the burden of AMR has reported the alarming statistic of 4.95 million deaths worldwide related to AMR in 2019 alone.^7^ The highest mortality rates were reported in Western and sub-Saharan Africa, at approximately 27.3 deaths per 100 000 individuals. The need to implement changes that combat and reduce AMR, both globally and in South Africa, is therefore urgent.^8^

Once infection has been confirmed, the use of antibiogram to guide treatment of susceptible organisms is recommended by the Society for Healthcare Epidemiology of America, the Infectious Diseases Society of America, and the Clinical and Laboratory Standards Institute (CLSI). Local susceptibility patterns should be determined such that appropriate empiric therapy can be utilised.^9^

This study aimed to determine the rate of culture positivity for uropathogens and the antimicrobial susceptibility patterns. This is to guide antimicrobial stewardship and prescribing practices at Potchefstroom Hospital; a regional hospital servicing the JB Marks municipality and drainage area in the North West province, South Africa. In addition, the results were compared with the National Department of Health’s standard treatment guidelines and National Essential Medicines List (NEML)^10^ for empiric antimicrobial management of confirmed UTIs.

The NEML currently recommends the following for treatment of an uncomplicated UTI^10^:

- Gentamicin 160 mg intramuscular as a single dose
- Fosfomycin 3 g po [per os, by mouth] as a single dose
- Nitrofurantoin 100 mg po four times a day for 5 days

Additionally, the NEML recommends the following for treatment of a complicated UTI^10^:

- Ciprofloxacin 500 mg po twice a day for 7–10 days

For pregnant women, the following has been recommended^10^:

- Fosfomycin 3 g po as a single dose
- Nitrofurantoin 100 mg po four times a day for 5 days

## Methods

### Ethical considerations

The research was approved by the Human Research Ethics Committee (Medical) of the University of the Witwatersrand (reference number: M230502 M230810-A-0011). Additionally, institutional approval was granted by the Potchefstroom Hospital Ethics Committee. Provincial approval was provided by the North West Department of Health, and National approval by the National Department of Health. Informed consent was not deemed necessary because of the absence of identifiable patient data and information being provided by the National Health Laboratory Service (NHLS) Academic Affairs and Research Management Systems for analysis. Data confidentiality was maintained in accordance with the Declaration of Helsinki and the Protection of Personal Information Act of 2013.

### Study design and setting

The study was a retrospective analysis of urine microscopy, culture and sensitivity specimens submitted to the NHLS at Potchefstroom Hospital. The NHLS in South Africa is a public entity providing diagnostic pathology services, research, and health surveillance, accredited by the South African National Accreditation System under ISO 15189 for medical laboratories. Potchefstroom Hospital is a state-funded, 335-bed regional healthcare facility in the Kenneth Kaunda district of South Africa’s North West province, offering specialised in- and outpatient care to patients in the surrounding area.

### Inclusion and exclusion criteria

The urine sample results included in the analysis were those submitted over 4 years between 01 January 2020 and 31 December 2023. All in- and outpatients from whom urine for microscopy, culture and sensitivity was collected were included. Samples that did not yield a positive microbial culture, cultures that did not have susceptibility testing, samples that had been rejected (for any reason) and cultures that exhibited mixed microbial growth were excluded. No formal prospective study sample size estimates were performed – all available isolates were included.

### Sampling

As this is a retrospective records review, the data were acquired from the NHLS Academic Affairs and Research Management Systems database, which functions as a central storage hub for all the primary information gathered by the NHLS and its subsidiaries. Once the access for the NHLS Academic Affairs and Research Management Systems data was granted, consultation with a data analyst and NHLS collaborator was utilised to ensure the validity of the data.

Since the Potchefstroom NHLS does not have the capability to culture samples independently, the results of the urine microscopy, culture and sensitivity and subsequent primary data were collated into the NHLS Academic Affairs and Research Management Systems from several sources. The majority of urine samples (*n* = 3137) were sent to an NHLS laboratory in Mafikeng for performance of culture. The laboratory at Tshepong Hospital analysed a further 371 samples, and the remaining 53 samples were sent to NHLS Charlotte Maxeke Johannesburg Academic Hospital for processing and reporting.

### Laboratory analysis

The testing facility processed the samples according to their local Standard Operating Procedure. MacConkey Agar and Blood agar plates are inoculated and aerobically incubated for 24 h at 35 °C – 37 °C. The standard practice is that if a specimen collected by voiding or catheterisation shows no growth after overnight incubation, it is reported as *no growth*. The specimen is then removed from the incubator and stored at room temperature, should further tests be requested by the treating doctors. If growth is detected on the plate, the colonies are counted and the sample provisionally resulted. Bacterial identification and antimicrobial susceptibility testing were carried out using the VITEK^®^ 2 automated system (bioMérieux, Marcy l’Ètoile, Rhône-Alpes, France). All antimicrobial susceptibility results were interpreted using the CLSI guidelines.^11^

The laboratory adheres to the standards set out in the CLSI guidelines. Compliance is ensured internally with numerous quality control programmes and externally with South African National Accreditation System audit.

### Statistical analysis

The data were entered into STATA, basic version 18.0 (StataCorp LLC, College Station, Texas, United States). Variables included in the analysis were the date the sample was received, the department of sample origin, the organism cultured, and the corresponding antibiotic susceptibility results. The frequency of each organism cultured, and the susceptibility profile of commonly used antimicrobials, were determined. Subsequently, the strength of association of antimicrobial efficacy against specific organisms was determined utilising the Chi-squared test and Fischer’s Exact Test (for *N* < 5), where a *p*-value < 0.05 was considered statistically significant.

The CLSI guidelines recommend that at least 30 isolates or organism-antibiotic combinations be available for inclusion into the report/analysis. As such, only the five most common organisms, namely *E. coli, K. pneumoniae, Proteus mirabilis, Enterococcus faecalis*, and *Enterococcus faecium*, consistently reached the threshold of 30 samples when combined over the study period.

The antimicrobials represented were selected based on current standard treatment guidelines, along with commonly used second-line agents. After assessing the susceptibility profile for each, the strength of the association was determined. Finally, we described the trends in susceptibility of these organisms over the study period 2020–2023.

## Results

**FIGURE 1:**
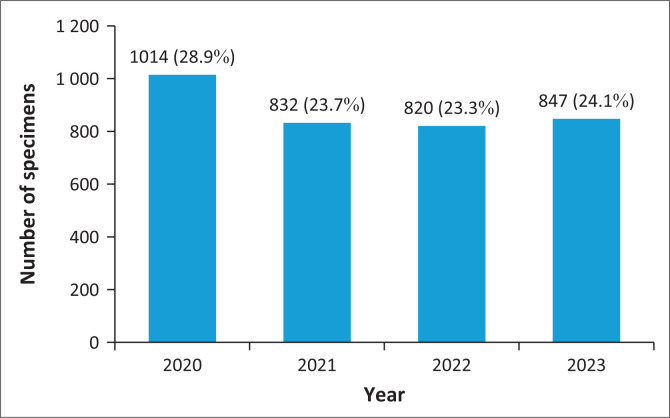
Number of positive urine cultures per year submitted from Potchefstroom Hospital, South Africa, January 2020 – December 2023.

A total of 3561 urine cultures were identified during the 4-year period, 48 of which were excluded from the final analysis because of the presence of more than one organism cultured. Therefore, a total of 3513 urine cultures were included in the data analysis. The number of positive urine cultures per year is depicted in Figure 1. During the study period, the number of samples that were positive for an organism decreased from 1014 samples in 2020 to an average of 833 per year for the following 3 years (832 for 2021; 820 for 2022; and 847 for 2023) (Figure 1).

**FIGURE 2:**
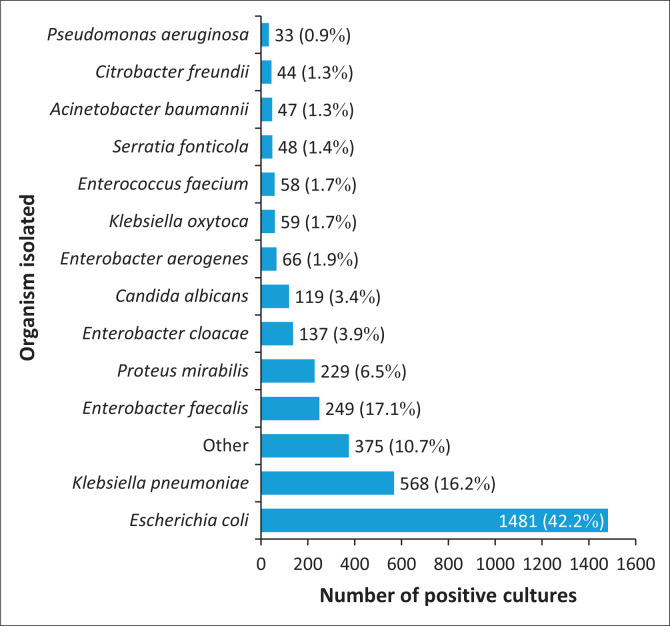
Spectrum of microorganisms isolated from urine samples submitted from Potchefstroom Hospital, South Africa, January 2020 – December 2023.

As outlined in Figure 2, the most frequently cultured organisms were *E. coli* (*n* = 1481; 42.2%), *K. pneumoniae* (*n* = 568; 16.2%), *E. faecalis* (*n* = 249; 7.1%), *P. mirabilis* (*n* = 229; 6.5%), *Enterobacter cloacae* (*n* = 137; 3.9%), and *Candida albicans* (*n* = 119; 3.4%).

**FIGURE 3:**
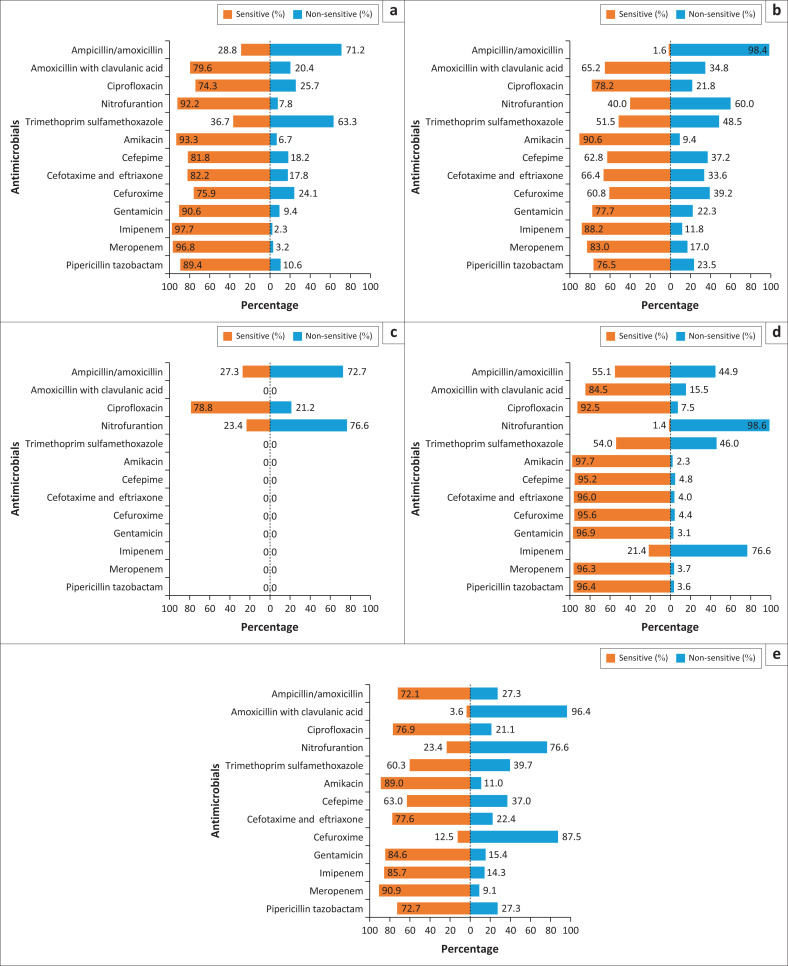
Antimicrobial susceptibility of the five most common uropathogens isolated at Potchefstroom Hospital, South Africa, January 2020 – December 2023: (a) *Escherichia coli*, (b) *Klebsiella pneumoniae*, (c) *Enterococcus faecalis*, (d) *Proteus mirabilis*, and (e) *Enterobacter cloacae*.

Regarding first-line antimicrobials (Table 1 and Figure 3), the aminoglycosides (gentamicin) would have been appropriate for 87.8% of the cultured organisms (*p <* 0.001), except *K. pneumoniae*, which was susceptible in only 77.7% of isolates. More than 90% of all organisms isolated were susceptible to amikacin (*p* = 0.001).

**TABLE 1:** Antimicrobial susceptibility patterns of the most commonly isolated organisms in urine specimens at Potchefstroom Hospital, South Africa, January 2020 – December 2023.

Antimicrobial	Susceptibility	*Enterobacter cloacae*		*Enterococcus faecalis*		*Escherichia coli*		*Klebsiella pneumoniae*		*Proteus mirabilis*		Total		χ^2^	*p*-value
		*n*	%	*n*	%	*n*	%	*n*	%	*n*	%	*n*	%		
**Amikacin**	Not sensitive	15	11.0	-	-	97	6.7	52	9.4	5	2.3	169	7.1	15.86	0.001
	Sensitive	121	89.0	-	-	1358	93.3	501	90.6	217	97.7	2197	92.9		
	Total	136	-	-	-	1455	-	553	-	222	-	2366	-		
**Amoxicillin with clavulanic acid**	Not sensitive	132	96.4	-	-	300	20.4	197	34.8	35	15.5	664	27.7	392.16	< 0.001
	Sensitive	5	3.6	-	-	1168	79.6	369	65.2	191	84.5	1733	72.3		
	Total	137	-	-	-	1468	-	566	-	226	-	2397	-		
**Cefepime**	Not sensitive	17	37.0	-	-	111	18.2	77	37.2	4	4.8	209	22.1	53.29	< 0.001
	Sensitive	29	63.0	-	-	498	81.8	130	62.8	80	95.2	737	77.9		
	Total	46	-	-	-	609	-	207	-	84	-	946	-		
**Cefotaxime and ceftriaxone**	Not sensitive	30	22.4	-	-	260	17.8	188	33.6	9	4.0	487	20.5	102.84	< 0.001
	Sensitive	104	77.6	-	-	1198	82.2	371	66.4	214	96.0	1887	79.5		
	Total	134	-	-	-	1458	-	559	-	223	-	2374	-		
**Gentamicin**	Not sensitive	21	15.4	-	-	138	9.4	126	22.3	7	3.1	292	12.2	84.55	< 0.001
	Sensitive	115	84.6	-	-	1336	90.6	438	77.7	221	96.9	2110	87.8		
	Total	136	-	-	-	1474	-	564	-	228	-	2402	-		
**Imipenem**	Not sensitive	19	14.3	-	-	33	2.3	65	11.8	176	78.6	293	12.5	1035.45	< 0.001
	Sensitive	114	85.7	-	-	1411	97.7	486	88.2	48	21.4	2059	87.5		
	Total	133	-	-	-	1444	-	551	-	224	-	2352	-		
**Meropenem**	Not sensitive	1	9.1	-	-	6	3.2	8	17.0	1	3.7	16	5.9	13.20	0.004
	Sensitive	10	90.9	-	-	179	96.8	39	83.0	26	96.3	254	94.1		
	Total	11	-	-	-	185	-	47	-	27	-	270	-		
**Pipericillin tazobactam**	Not sensitive	3	27.3	-	-	20	10.6	12	23.5	1	3.6	36	12.9	10.14	0.017
	Sensitive	8	72.7	-	-	168	89.4	39	76.5	27	96.4	242	87.1		
	Total	11	-	-	-	188	-	51	-	28	-	278	-		
**Cefuroxime**	Not sensitive	119	87.5	-	-	355	24.1	221	39.2	10	4.4	705	29.4	335.83	< 0.001
	Sensitive	17	12.5	-	-	1119	75.9	343	60.8	217	95.6	1696	70.6		
	Total	136	-	-	-	1474	-	564	-	227	-	2401	-		
**Ampicillin / Amoxicillin**	Not sensitive	8	72.7	14	6.5	1050	71.2	556	98.4	102	44.9	1730	69.4	Fisher’s Exact Test used	< 0.001
	Sensitive	3	27.3	202	93.5	424	28.8	9	1.6	125	55.1	763	30.6		
	Total	11	-	216	-	1474	-	565	-	227	-	2493	-		
**Trimethoprim sulfamethoxazole**	Not sensitive	52	39.7	18	100.0	928	63.3	271	48.5	104	46.0	1373	57.2	81.38	< 0.001
	Sensitive	79	60.3	0	0.0	537	36.7	288	51.5	122	54.0	1026	42.8		
	Total	131	-	18	-	1465	-	559	-	226	-	2399	-		
**Ciprofloxacin**	Not sensitive	29	21.2	6	23.1	378	25.7	123	21.8	17	7.5	553	22.8	37.72	< 0.001
	Sensitive	108	78.8	20	76.9	1093	74.3	440	78.2	210	92.5	1871	77.2		
	Total	137	-	26	-	1471	-	563	-	227	-	2424	-		
**Nitrofurantoin**	Not sensitive	98	76.6	1	100.0	111	7.8	327	60.0	217	98.6	754	32.5	1140.39	< 0.001
	Sensitive	30	23.4	0	0.0	1317	92.2	218	40.0	3	1.4	1568	67.5		
	Total	128	-	1	-	1428	-	545	-	220	-	2322	-		

The cumulative susceptibility to nitrofurantoin was 67.5% (*p* < 0.001). Of the five most commonly isolated organisms, *E. coli* isolates were the only susceptible organism (92.2%). Other isolates, such as *K. pneumoniae* (40%), *E. cloacae* (23.4%) *P. mirabilis* (1.4%), *E. faecalis* (0%), all had reduced susceptibility to nitrofurantoin.

A similar trend of emerging resistance to ciprofloxacin was observed in four of the five most frequently isolated pathogens in this study; *P. mirabilis* remained the only persistently sensitive organism (92.5%), while reduced susceptibility was detected in *E. cloacae* (78.8%), *K. pneumoniae* (78.2%), *E. faecalis* (76.9%), and *E. coli* (74.3%). Overall, organisms had a cumulative sensitivity of 77.2% (*p* < 0.001).

Individual sensitivity to trimethoprim sulfamethoxazole were as follows: *E. cloacae* (60.3%), *P. mirabilis* (54%), *K. pneumoniae* (51.5%), *E. coli* (36.7%), and *E. faecalis* (0%). Cumulatively, sensitivity to trimethoprim sulfamethoxazole was 42.8% (*p* < 0.001).

Significant resistance to amoxicillin/clavulanic acid was present in *E. cloacae* (3.6% susceptible), *K. pneumoniae* (65.2% susceptible), and *E. coli* (79.6% susceptible), with 72.3% of all organisms isolated being susceptible (*p* < 0.001). There was a similar pattern with ampicillin, with an overall susceptibility of 30.6% of all the cultured organisms (*p* < 0.001). The susceptibility of individual organisms to amoxicillin/clavulanic acid was also greatly reduced, namely, *K. pneumoniae* (1.6%), *E. cloacae* (27.3%), *E. coli* (28.8%), and *P. mirabilis* (55.1%). Ampicillin showed continued sensitivity of 93.5% for *E. faecalis*.

For second-line antimicrobials, the cumulative susceptibility of the organisms to cefuroxime was 70.6% (*p* < 0.001), cefotaxime/ceftriaxone (79.5%; *p* < 0.001), and cefepime (77.9%; *p* < 0.001) (Table 1). The third- and fourth generation cephalosporins were effective against *P. mirabilis*, with sensitivities as high as 96% (third generation) and 95.2% (fourth generation). The sensitivity of *E. coli* to third generation cephalosporins was 82.2%, and to fourth generation, 81.8% and 66.4% (third generation) and 62.8% (fourth generation) for *K. pneumoniae*. Similar rates of resistance were seen in *E. cloacae*, with susceptibilities of 77.6% (third generation) and 63% (fourth generation).

The overall cumulative sensitivity to meropenem was 94.1% (*p* = 0.004), and 87.5% (*p* < 0.001) to imipenem. *Proteus mirabilis* was only sensitive in 96.3% of cases to meropenem, and to imipenem in 21.4% of cases. *E. coli, K. pneumoniae* and *E. cloacae* had sensitivities of over 83% to bothimipenem and meropenem.

The cumulative sensitivity to piperacillin–tazobactam was 87.1% (*p* = 0.017). *Escherichia coli* was relatively sensitive, at 89.4%, as was *P. mirabilis*, at 96.4%, whereas *K. pneumoniae* (76.5%) and *E. cloacae* (72.7%) both had reduced sensitivity.

*Candida albicans* isolates were, as expected, relatively sensitive to fluconazole (92.9%; *p* < 0.001), amphotericin B (92.9%; *p* < 0.001) and voriconazole (94.9%; *p* < 0.001) (Table 3).

During the study period, *Pseudomonas aeruginosa* was isolated on 33 occasions. Susceptibility testing reached the threshold of 30 or more isolates for four antibiotics: amikacin (sensitivity 97%), gentamicin (sensitivity 50%), ciprofloxacin (sensitivity 76%), and piperacillin–tazobactam (sensitivity 94%) (Table 2). *Acinetobacter baumannii* was isolated 47 times, but only three antibiotics were tested in 30 or more isolates. These included ciprofloxacin (37% sensitivity), gentamicin (42% sensitivity), and piperacillin–tazobactam (28% sensitivity) (Table 2).

**TABLE 2:** Antimicrobial susceptibility patterns of *Pseudomonas aeruginosa* and *Acinetobacter baumannii* in urine specimens at Potchefstroom Hospital, South Africa, January 2020 – December 2023.

Antimicrobial	Susceptibility	*Pseudomonas aeruginosa*		*Acinetobacter baumannii*	
		*n*	%	*n*	%
**Amikacin**	Sensitive	32	96.97	-	-
	Not sensitive	1	3.03	-	-
	Total	33	-	-	-
**Gentamicin**	Sensitive	32	50.00	19	42.22
	Not sensitive	32	50.00	26	57.78
	Total	64	-	45	-
**Ciprofloxacin**	Sensitive	25	75.76	17	36.96
	Not sensitive	8	24.24	29	63.04
	Total	33	-	46	-
**Pipericilin with tazobactam**	Sensitive	29	93.55	12	27.91
	Not sensitive	2	6.45	31	72.09
	Total	31	-	43	-

**TABLE 3:** Antimicrobial susceptibility patterns of *Candida albicans* species in urine specimens at Potchefstroom Hospital, South Africa, January 2020 – December 2023.

Antimicrobial	Susceptibility	*Candida albicans*		*p*-value
		*n*	%	
**Amphotericin B**	Not sensitive	8	7.08	< 0.001
	Sensitive	105	92.92	
	Total	113	100	
**Fluconazole**	Not sensitive	8	7.02	< 0.001
	Sensitive	106	92.92	
	Total	114	100	
**Voriconazole**	Not sensitive	3	5.08	< 0.001
	Sensitive	56	94.92	
	Total	59	100	

There were significant changes in trend noted for second-, third-, and fourth generation cephalosporins, and nitrofurantoin over the study period (Table 4). The organisms demonstrated an increase in sensitivity to cefepime, rising from 73.6% to 78.6% (*p* = 0.033). In contrast, sensitivity to cefotaxime/ceftriaxone and cefuroxime declined over the study period, decreasing from 79.5% to 75.6% (cefotaxime/ceftriaxone; *p* = 0.006) and from 68.7% to 63.9% (cefuroxime; *p* = 0.009). Sensitivity to nitrofurantoin also improved over the study period, from 32.8% in 2020 to 37.2% by 2023 (*p* = 0.013).

**TABLE 4:** Trend in cumulative antimicrobial susceptibility rates at Potchefstroom Hospital, South Africa, January 2020 – December 2023.

Antimicrobial	2020		2021		2022		2023		*p*-value
	Sensitive	Resistant	Sensitive	Resistant	Sensitive	Resistant	Sensitive	Resistant	
Ampicillin / Amoxicillin	560	217	462	231	474	181	440	182	NS
Amoxicillin with clavulanic acid	573	573/238	461	210	442	225	448	203	NS
Cefuroxime	555	253	483	183	459	209	419	237	0.009
Cefotaxime / Ceftriaxone	645	166	562	111	528	140	481	155	0.006
Cefepime	315	113	281	64	187	43	92	25	0.033
Imipenem	701	104	602	602	584	85	538	98	NS
Meropenem	88	6	90	5	306	21	198	15	NS
Piperacillin with tazobactam	113	23	102	19	90	19	58	16	NS
Amikacin	762	67	633	50	648	34	595	48	NS
Gentamicin	734	107	628	73	599	95	584	95	NS
Ciprofloxacin	659	197	564	144	549	154	510	175	NS
Trimethoprim sulfamethoxazole	452	391	372	328	384	300	364	300	NS
Nitrofurantoin	261	535	174	436	221	442	238	401	0.013

NS, not significant.

## Discussion

The prevalence of the various uropathogens identified in this study aligns with findings from a recent investigation at RK Khan Hospital in KwaZulu-Natal, South Africa, which employed a similar sample size and methodology. In this study, *E. coli* was the predominant pathogen, followed by *K. pneumoniae* and *E. faecalis*.^12^

The authors of the RK Khan Hospital study found that resistance rates to nitrofurantoin were low in *E. coli*, at 6.2%, and high in *K. pneumoniae*, at 61.3%.^12^ These figures reflect our findings. A multicentre local study conducted by Lewis et al. in 2013 forms part of the basis of the NEML, in which good cumulative sensitivity to nitrofurantoin (91.7%) was noted.^13^ The markedly reduced sensitivities of *K. pneumoniae* (40%), *E. cloacae* (23.4%), and *P. mirabilis* (1.4%) highlight emerging resistance and make nitrofurantoin less useful in our setting for empiric treatment. Additionally, the CLSI states that *P. mirabilis* is intrinsically resistant to nitrofurantoin, which reflects our results.^14^

Given that resistance patterns tend to differ by geographical location, and the current recommendations by the NEML are based partially on American data, we believe it was necessary to verify the NEML recommendations for the use of gentamicin. The suggested utilisation of gentamicin in the guidelines was based on a systematic review, published in 2018, in Arizona (United States) which showed that 94.5% of pathogens isolated from pooled studies were susceptible to gentamicin.^15^ At a local level, Naidoo et al. also noted a lower cumulative susceptibility of 65% in *K. pneumoniae*.^12^ In this study, *K. pneumoniae* was sensitive to gentamicin in 77.7% of isolates, a 12.7% higher sensitivity rate when compared to Naidoo et al. The use of gentamicin may still be an effective empiric antimicrobial in our setting, as this study revealed an 87.8% cumulative sensitivity overall.

The Infectious Diseases Society of America guidelines recommend that ciprofloxacin be used as empiric therapy for complicated UTIs, only if resistance rates are below 10%.^16^ Lewis et al. noted that cumulative sensitivity to fluoroquinolones (94.1%) had been maintained; however, Naidoo et al. published alarming ciprofloxacin sensitivity rates, with only 62% of *E. coli* and 70% of *Klebsiella* spp. found to be sensitive.^12,13^ The authors highlighted that the reduced sensitivity would lead to high treatment failure rates with empiric ciprofloxacin use.^12^ Another study conducted at 3 Military Hospital (Bloemfontein) in 2011 also demonstrated that cultures in complicated UTIs showed a reduced sensitivity to ciprofloxacin (41%),^17^ and the authors recommended against its use at that hospital.^17^

Ciprofloxacin has been reported to promote resistance through horizontal transmission of resistance genes. contributing collateral damage with higher resistance rates in other classes of antimicrobials.^17,18^ Emerging resistance to ciprofloxacin was noted in four of the five common pathogens in the current study, specifically *E. cloacae* (79%), *K. pneumoniae* (78%), *E. faecalis* (77%) and *E. coli* (74%), with *P. mirabilis* remaining susceptible, at 92.5%. Ciprofloxacin was once the preferred treatment for UTIs, but this is no longer the case in our setting.

Lewis et al. also reported reduced cumulative sensitivity rates of 82.8% to amoxicillin–clavulanate in 2013,^13^ and similarly, Naidoo et al. found low sensitivity in both *K. pneumoniae* (58%) and *E. coli* (70%).^12^ A high level of resistance towards amoxicillin clavulanate, with reduced cumulative sensitivity of 72.3%, was also noted in our study, indicating that these agents may not be effective towards the two most prevalent uropathogens, namely *E. coli* and *K. pneumoniae*. Whereas Lewis et al. reported a high rate of sensitivity to cefuroxime (93.4%) in 2013,^13^ Naidoo et al. showed that sensitivity to third-generation cephalosporins was only 74% in *E. coli*, and 58% in *K. pneumoniae*, which correlated with the reduced sensitivities in this study.^12^ The inherent resistance to cephalosporins found in *E. faecalis* has led the NHLS to forgo susceptibility testing to cephalosporins.^19^

*Enterobacter cloacae* has been reported to be intrinsically resistant to amoxicillin–clavulanate by the CLSI, which is in line with our study (96.4%).^14^ Furthermore, this organism is an AmpC-producer and is therefore at high risk of developing AmpC enzyme overproduction, subsequently leading to third-generation cephalosporin resistance.^14^ Although we have variable sensitivities in our beta-lactams, CLSI recommend against the use of third-generation cephalosporins and piperacillin–tazobactam because of the risk of treatment failure.^14^

Naidoo et al. demonstrated meropenem sensitivities of 95% for *K. pneumonia* and 100% for *E. coli*.^12^ These findings mirrored the cumulative sensitivity of our study, of 94.1% overall. Similarly, the authors reported the sensitivity to piperacillin–tazobactam to be 76% for *K. pneumoniae* and 95% for *E. coli*, which is consistent with our findings of 76.5% for *K. pneumoniae* and 89.4% for *E. coli*.^12^

There was a reduced cumulative sensitivity to trimethoprim–sulfamethoxazole at 42.8%. These figures were mirrored by Lewis et al., with a cumulative sensitivity of 44.3%, and Naidoo et al., with 39% *E. coli* and 52% *K. pneumoniae* susceptibility.^12,13^ The Infectious Diseases Society of America guidelines recommend the use of trimethoprim–sulfamethoxazole for empiric therapy for uncomplicated UTIs only if local uropathogen resistance rates do not exceed 20%.^16^ In our setting, and considering sensitivities from other local studies, trimethoprim–sulfamethoxazole would unlikely be suitable as an empiric agent.^12,15,17^

Fosfomycin susceptibility testing is not routinely performed by NHLS North West, and thus no analysis was performed.

Future therapies include the possibility of using vaccines to prevent recurrent UTIs. A meta-analysis by Mak et al. in 2024, showed limited evidence for this approach, but that more rigorous research is needed in order to determine the value of vaccines and oral bacterial lysates immunostimulants as an option for prophylactic treatment.^20^

We recommend that *E. coli* be considered as the culprit pathogen in both in-and outpatient UTIs and, in compliance with most international guidelines, nitrofurantoin should be used as first-line agent. This does not negate the need for culture and sensitivity to be performed, as well as de-escalation or escalation of therapy, as needed. If parenteral therapy is necessary, most organisms remain susceptible to gentamicin, and although we do not have data for fosfomycin at Potchefstroom Hospital, other local data support its use for empiric therapy.

### Limitations

This study was a retrospective analysis and some of the samples were processed at various sites which could have affected the results.

While the study has a significant sample size, the data was collected from a single site and is only truly descriptive of the microbiome at Potchefstroom Hospital, and these findings may not be generalisable to other institutions or to the broader population.

Similarly, data were collected from patients and samples at a regional hospital and, therefore, may not be fully representative of the type of infections that would be encountered in the primary healthcare setting. Furthermore, Carbapenemase-resistant Enterobacterales and Extended Spectrum Beta-lactamase organisms were not analysed, as it was beyond the scope of our article. Further research is recommended to evaluate the prevalence and morbidity of these organisms.

Being a laboratory-based study, demographic and clinical data were not collated and as such, we cannot make recommendations with regard to tailoring of antimicrobial therapy to specific clinical scenarios. In addition, it was not possible to differentiate between organism colonisation or contamination, asymptomatic bacteriuria, and true infection, and whether they were community- or hospital-acquired isolates.

### Conclusion

In this study, we have determined the prevalence of the most common uropathogens isolated from samples submitted to Potchefstroom Hospital. *Escherichia coli* remains the most prevalent uropathogen; however, our findings show that *E. coli* and most other organisms isolated from urine samples at Potchefstroom Hospital were not susceptible to the first-line antimicrobial agents as suggested by the NEML. Ciprofloxacin is not recommended as the first-line agent for any UTI at Potchefstroom Hospital, unless susceptibility is proven.

This study also provides some recommendations for second- and third-line antimicrobials while awaiting susceptibility testing. In general, the other recommended first-line antimicrobial agents, gentamicin, nitrofurantoin, and fosfomycin are likely to treat most UTIs empirically. Given the high rate of drug-resistant uropathogens, careful selection of empiric therapy is vital to ensure acceptable clinical outcomes and to prevent further development of AMR. This study has provided an updated antibiogram for uropathogens isolated at Potchefstroom Hospital in the North West province.
